# Clinical, mycological, and antifungal susceptibility testing of causative agents of fungal keratitis from North India

**DOI:** 10.22034/cmm.2025.345456.1692

**Published:** 2026-02-01

**Authors:** Ankita Saroya, Amit Gupta, Chintan Malhotra, Arpita Khamrai, Snigdha Reddy, Shivaprakash M Rudramurthy, Harsimran Kaur, Anup Ghosh

**Affiliations:** 1 Advanced Eye Centre, Postgraduate Institute of Medical Education and Research (PGIMER), Chandigarh, India; 2 Department of Medical Microbiology, Postgraduate Institute of Medical Education and Research (PGIMER), Chandigarh, India

**Keywords:** Fungal keratitis, Antifungal Susceptibility testing, Luliconazole, Epidemiological Cut off, Clinical and Laboratory Standards Institute

## Abstract

**Background and Purpose:**

Fungal Keratitis (FK) is a sight-threatening corneal infection resulting in monocular blindness. FK occurs more frequently in warm, humid tropical and subtropical regions, particularly those with an agriculture-based economy. The present study aims to check the spectrum of organisms causing FK and analyze the associated clinical features. Antifungal susceptibility patterns also determined with isolated organisms in a tertiary care center in North India.

**Materials and Methods:**

This study was conducted on suspected FK cases recruited from January 2020 through December 2023 in the Advanced Eye Centre (AEC) Postgraduate Institute of Medical Education and Research (PGIMER), Chandigarh. The routine mycological laboratory data, including direct microscopy (KOH mount) and culture on Sabouraud’s Dextrose agar media (SDA), were collected. The clinical data and etiological factors were analysed by retrieving medical records. Further, the preserved isolates were subjected to antifungal susceptibility testing as per the Clinical and Laboratory Standards Institute (CLSI) broth microdilution method (M38-A2) for a panel of antifungals, including VRCZ (voriconazole), ITRA (itraconazole), PSCZ (posaconazole), LLCZ (luliconazole), MCZ (miconazole), KTZ (ketoconazole), AMB (amphotericin-B), NTM (natamycin), and TFH (terbinafine-hydrochloride).

**Results:**

Of a total of 2891 suspected cases, 677 were found to be positive by either microscopy or culture. Of the 677 positive cases, 470 were culture-positive, 383 (56.57%) were both microscopy as well as culture-positive, 206 (30.42%) were culture-negative but microscopy-positive, and 87 (12.85%) were culture-positive but microscopy-negative. In the geographical zones from , Punjab was leading with 39.29% of cases, followed by Haryana (26.14%), Uttar Pradesh (14.33%), and Himachal Pradesh (14.03%). *Aspergillus* spp. was found to be the predominant etiologic agent accounting for (N=198/470) 42.12%, followed by *Fusarium* spp. (N=173/470) 36.80%, dematiaceous fungi (N=71/470) 15.10%. Clinical details of all 677 patients were successfully retrieved and found that vegetative trauma was the major associated etiological factor causing FK. Other etiological factors involved animal tails, dust, insects, foreign bodies, chemicals, or iron piece injuries. AFST results revealed that VRCZ, ITRA, and LLCZ exhibit low MIC values against *Aspergillus* spp. and *Fusarium* spp. VRCZ exhibits a median MIC range of 0.25µg/ml among Aspergillus spp. and 0.5µg/ml among Fusarium spp. and the dematiaceous group of fungi.

**Conclusion:**

*Aspergillus* spp. represents the predominant etiology causing FK, followed by *Fusarium* spp., but dematiaceous fungi seem to pose a significant threat in causing FK. In-vitro susceptibility results revealed that with VRCZ, LLCZ can also be a promising antifungal drug for the treatment of FK.

## Introduction

Infectious keratitis is an inflammation in the cornea caused by bacteria, fungi, parasites, or viruses, and the fifth leading cause of corneal blindness globally [1,2]. Fungal keratitis (FK) is estimated to be over a million cases annually [3]. It is a serious, sight-threatening infection of the cornea with few effective therapies available, which frequently result in poor outcomes [4]. There was a country-wise variation in prevalence, ranging from 50.1% in Paraguay to 1.1% in Ireland [5]. Countries, such as China, Egypt, Ethiopia, Ghana, and Paraguay, report a high percentage of the FK cases, indicating a significant regional burden of the disease [6]. In India, the prevalence of FK ranges from 21% to 37% of all the infective keratitis cases (Statpearls, NCBI, n.d.). Paty et al. (2018) reported that the prevalence of FK ranges from 25.6% to 36.7% in India [7]. A higher prevalence of FK was observed in South India, at 36.7%, followed by 36.3% in western India, and 7.3-25.6% in North and Northeast India [8].

*Aspergillus* spp. and *Fusarium* spp. are the most predominant causative agents, followed by the dematiaceous group of fungi for FK in India [8]. In the dematiaceous group, especially *Curvularia* sp. was most common for FK, followed by *Alternaria alternata*, *Exseorhilum rostratum*, *Scedosporium* sp., and *Bipolaris* sp. [9,10]. Although there is a change in the spectrum of organisms and many newer species are included in the list, an effective antifungal treatment is still warranted. Commonly used antifungal agents against FK include azoles, such as voriconazole (VRCZ), ketoconazole (KTZ), posaconazole (PSCZ), and itraconazole (ITRA). Among the polyenes, natamycin (NTM) is the drug of choice, while amphotericin B (AMB) is used for the treatment of severe cases of FK. A new imidazole, luliconazole (LLCZ), is being reported to have better *in vitro* efficacy against *Aspergillus* spp. and *Fusarium* spp., but is still not used for the treatment of FK patients due to the lack of limited studies available [11]. The *in vitro* antifungal susceptibility interpretation may be helpful for ophthalmologists to choose the optimal therapy for the treatment of FK patients [12]. With the rising incidence and changing spectrum of fungal species causing FK, there is a lack of comprehensive data on the *in vitro* susceptibility of these emerging pathogens, especially newer agents, like LLCZ. Given this background, the present study aimed to analyse the spectrum of organisms, the clinical presentation, and the *in vitro* antifungal susceptibility pattern of FK isolates from January 2020 to December 2023 at a tertiary care centre in North India.

## Materials and Methods

### Study Design

A study was conducted on all clinically suspected FK patients who attended the Advanced Eye Centre at the Postgraduate Institute of Medical Education and Research (PGIMER), Chandigarh, from January 2020 to December 2023. Ethical approval was obtained from the Institutional Ethics Committee, PGIMER (Ref. No. IEC-02/2021-1890) .

Patients were included in the study if they had a relevant history and were clinically suspected of having FK Patients with laboratory-confirmed bacterial or viral keratitis were excluded.

### Clinical and mycological examination

Patients presenting with suspected FK underwent investigation through slit-lamp microscopy by an ophthalmologist. Diagnosis of FK was based on clinical criteria, including history and characteristic features, such as corneal ulceration with feathery margins, dry and grey elevated infiltrates, the presence of satellite lesions, epithelial defect, and hypopyon, along with mycological criteria. Corneal samples, including scraping and buttons, were collected under aseptic conditions and sent to the mycology laboratory for microscopy and culture. Part of the sample used for microscopy underwent Gram staining, a 10% potassium hydroxide (KOH) mount, and a calcofluor white wet mount. The other part of the sample was inoculated in a ‘C’ shape on Sabouraud dextrose agar with Chloramphenicol (Hi-Media), as well as on blood agar plates, and incubated Sabouraud dextrose agar plates for up to two weeks at 25 °C, respectively, to observe for fungal growth .

Clinical data, including detailed patient histories along with socio-demographic features, duration of symptoms, etiological factors, slit lamp findings, and associated ocular conditions, were retrieved from the medical records of the Advanced Eye Centre, PGIMER. All microscopy and culture positivity data were collected from the mycology laboratory at PGIMER.

### In vitro antifungal susceptibility testing

Preserved isolates were retrieved from the National Culture Collection of Pathogenic Fungi. The antifungal susceptibility testing (AFST) was prospectively performed by broth microdilution assay following the Clinical and Laboratory Standards Institute (CLSI) M38-A2 (for filamentous fungi) [13]. A panel of nine antifungals was selected, namely VRCZ, ITRA, PSCZ, KTZ, MCZ, LLCZ, AMB, NTM, and Terbinafine hydrochloride (TFH) (Sigma Aldrich, Bangalore, India). The antifungals were serially two-fold diluted to 10 concentrations in Rosewell Park Memorial Institute 1640 medium (RPMI 1640) buffered with MOPS (pH 7.0). The drugs were dissolved in dimethyl sulfoxide, and the final concentrations were within the ranges of 0.03-16µg/ml for VRCZ, ITRA, PSCZ, KTZ, MCZ, and AMB; 0.001-0.5µg/ml for LLCZ; 0.06-32µg/ml for NTM; and 0.12-32µg/ml for TFH. The drug plates prepared initially contained 2× concentration of drugs in 100 µL RPMI 1640 in 96-well round-bottom microtiter plates. The consequent addition of 100 µL inoculum diluted the drug to the required (CLSI-prescribed) concentration. The inoculum was prepared by harvesting fungal spores by washing of fungal mycelia with sterile 0.85% normal saline. The fungal spore suspensions were diluted 50-fold in RPMI 1640 to attain a spore suspension at a final concentration of 0.4-5 × 10^4^ CFU/ml. The plates were incubated at 28 °C for 48-72 h (*Aspergillus* and *Fusarium*) or until visible growth was observed in the growth control, and allowed for accurate endpoint determination in cases of dematiaceous fungi. The minimum inhibitory concentration (MIC) determined was the lowest concentration of drug exhibiting 100% inhibition of any visually recognizable fungal growth [9]. *Aspergillus flavus* ATCC 204304 was used as a reference strain. Interpretation of the AFST was done following CLSI M59 and CLSI M61 recommendations, by referring to the clinical breakpoints or epidemiological cut-off values (ECVs), wherever necessary [14].

### Statistical analysis

Descriptive statistics were used for analysis, summarizing the sociodemographic, clinical characteristics, microscopic, and culture results [15]. The analysis was carried out using the Statistical Package for the Social Sciences software (SPSS Inc., Chicago, IL, version 23.0 for Windows). The proportions were compared using the Chi-square test (χ^2^) or Fisher’s exact test when applicable, and a *P* value of ≤ 0.05 was considered significant.

## Results

A total of 2,891 suspected FK patients attended the Advanced Eye Centre, PGIMER, between January 2020 through December 2023. However, the complete medical records could be retrieved for only 677 patients, who were used for the detailed analysis. Among them, 593 (87.59%) were microscopy-positive and 470 (69.42%) were culture-positive. There were 383 cases with both microscopy as well as culture positivity (56.57%), 206 culture-negative but microscopy-positive cases (30.42%), and 87 culture-positive but microscopy-negative cases (12.85%).

### Demographics and clinical characteristics

Punjab is the leading geographical zone with a record of 266 (39.29%) patients, followed by Haryana, Uttar Pradesh, and Himachal Pradesh, with 177 (26.14%), 97 (14.32%), and 95 (14.03%) cases, respectively (Figure 1). Demographic data revealed the age group ranged from 1 to 87 years, with a mean and median of 49 years and 50 years.

**Figure 1:**
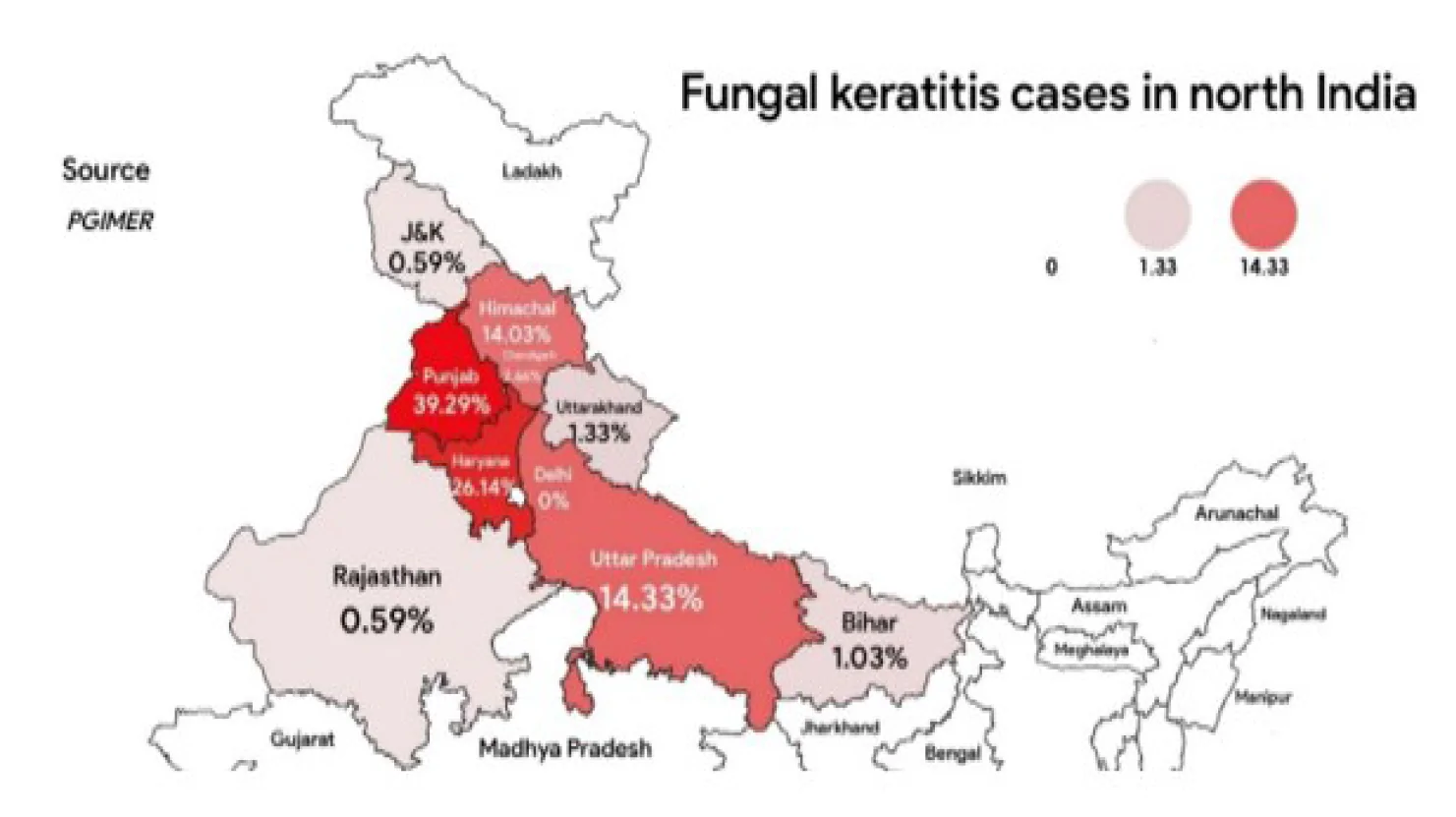
Regional distribution of fungal keratitis cases

Clinical presentations of recorded FK patients (Figure 2) revealed that the chief complaint was redness in the eye, which was observed in patients with *Aspergillus* spp. (n=128) 64.64% followed by *Fusarium* spp. (n=105) 60.69%, and *dematiaceous* fungi (n=43) 60.56%. Etiological factors involved (Table 1) the vegetative trauma at the utmost (n=108), 54.54% of which were predominantly caused by *Aspergillus* spp.; exposure to animal tails, dust, insects, foreign bodies, and chemical or iron piece injuries were also reported.

**Figure 2:**
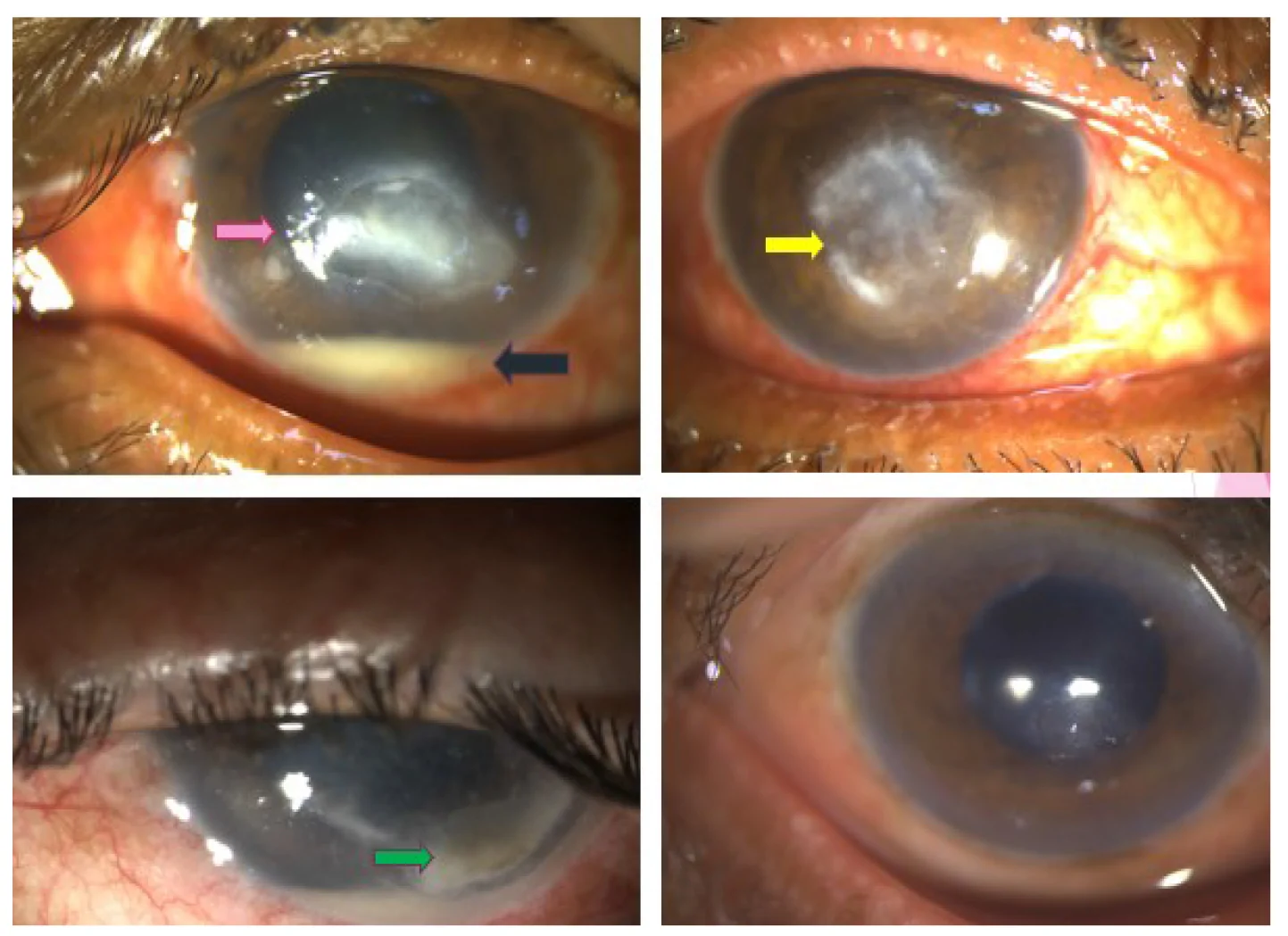
Slit lamp microscopic examination images of patients with fungal keratitis. a) *Aspergillus flavus* infected FK, purple arrow showing a white patchy appearance and blue arrow showing the presence of hypopyon, b) *Fusarium* spp. infected FK, yellow arrow showing a feathery and patchy appearance, c) *Dematiaceous* fungi (*Exserohilum rostratum*) infected FK, green arrow showing feathery appearance and pigmentation, d) healed eye of *Dematiaceous*-infected FK

**Table 1:** Detailed aetiology-wise analysis and clinical features among the three major groups of *Aspergillus*, *Fusarium*, and Dematiaceous fungi-infected FK patients

		*Aspergillus* spp. (n=198)	*Fusarium* spp. (n=173)	Dematiaceous fungi (n=71)	*p* value
**Etiological Factors**	Vegetative matter	108 (54.5%)	31(17.9%)	17 (23.9%)	<0.001
	Animal Tail	1 (0.5%)	1 (0.5%)	0	0.82
	Dust	3 (1.5%)	5 (2.8%)	4 (5.6%)	1.84
	Insect	3 (1.5%)	3 (1.7%)	1 (1.4%)	0.978
	Foreign body	24 (12.1%)	29 (16.7%)	11 (15.4%)	0.432
	Chemical injury	2 (1.0%)	1 (0.5%)	0	0.659
	Iron piece injury	2 (1.0%)	2 (1.1%)	1 (1.4%)	0.963
**Clinical presentation**	Pain	67 (33.8%)	64 (36.9%)	23 (32.3%)	0.730
	Redness	128 (64.6%)	105 (60.6%)	43 (60.5%)	0.690
	Watering	59 (29.7%)	51 (29.4%)	24 (33.8%)	0.782
	Diminished vision	58 (29.2%)	81 (46.8%)	26 (36.6%)	0.002
	Hypopyon	66 (33.3%)	35 (20.2%)	20 (28.1%)	---
	Duration of symptoms	27.37 days (average)	28.63 days (average)	21 days (average)	---

### Mycological findings

*Aspergillus* spp. was the predominant agent, accounting for 198 (42.12%) cases with species of *A. flavus* (n=167/470, 35.53%), *Aspergillus fumigatus* (n=23/470, 4.89%), *Aspergillus terreus* (n=5/470, 1.06%), and *Aspergillus versicolor* (n=1/470, 0.21%). The other clinically significant isolates include *Fusarium* spp. (n=173/470; 36.80%) and *Dematiaceous* fungi (n=71/470; 15.10%). However, yeasts (n=21/470; 4.46%) and other moulds (n=7/470; 1.48%) were also isolated (Table 2). Year-wise analysis revealed that 2023 had the highest distribution of significant etiological agents (Figure 3).

**Table 2:** Spectrum of organisms isolated from corneal samples

Fungal species		Incidence (n=470)	Percentage
*Aspergillus* spp.	*Aspergillus flavus*	n=167	36%
	*Aspergillus fumigatus*	n=23	3.89%
	*Aspergillus terreus*	n=5	1.06%
	*Aspergillus nidulans*	n=2	0.42%
	*Aspergillus versicolor*	n=1	0.21%
*Fusarium* spp.	*Fusarium* spp.	n=117	25%
	*Fusarium* solani	n=33	7.02%
	*Fusarium* incarnatum	n=2	0.42%
	*Fusarium proliferatum*	n=6	1.27%
	*Fusarium oxysporum*	n=6	1.27%
	*Fusarium sacchari*	n=1	0.21%
	*Fusarium temperatum*	n=1	0.21%
	*Fusarium falciforme*	n=2	0.42%
	*Fusarium verticillioides*	n=3	0.63%
	*Fusarium daphnoides*	n=2	0.42%
*Curvularia* spp.	*Curvularia* spp.	n=6	1.27%
	*Curvularia lunata*	n=26	5.6%
	*Curvularia geniculata*	n=5	1.06%
	*Curvularia clavata*	n=1	0.21%
	*Curvularia spicifera*	n=1	0.21%
	*Corynespora cassiicola*	n=1	0.21%
*Bipolaris* sp.		n=4	0.85%
*Alternaria* sp.	n=8	1.07%
*Exserohilum rostratum*		n=5	1.06%
*Scedosporium* sp.	n=2	0.42%
*Colletotrichum* sp.		n=4	0.85%
*Lasiodiplodia* sp.	n=6	1.27%
*Cladosporium* sp.		n=1	0.21%
*Epicoccum nigrum*	n=1	0.21%
*Candida* spp.	*Candida albicans*	n=10	2.12%
	*Candida parapsilosis*	n=6	1.27%
	*Candida krusei*	n=2	0.42%
	*Candida kefyr*	n=1	0.21%
	*Candida tropicalis*	n=2	0.42%
Other molds	Other molds	n=7	1.48%

**Figure 3:**
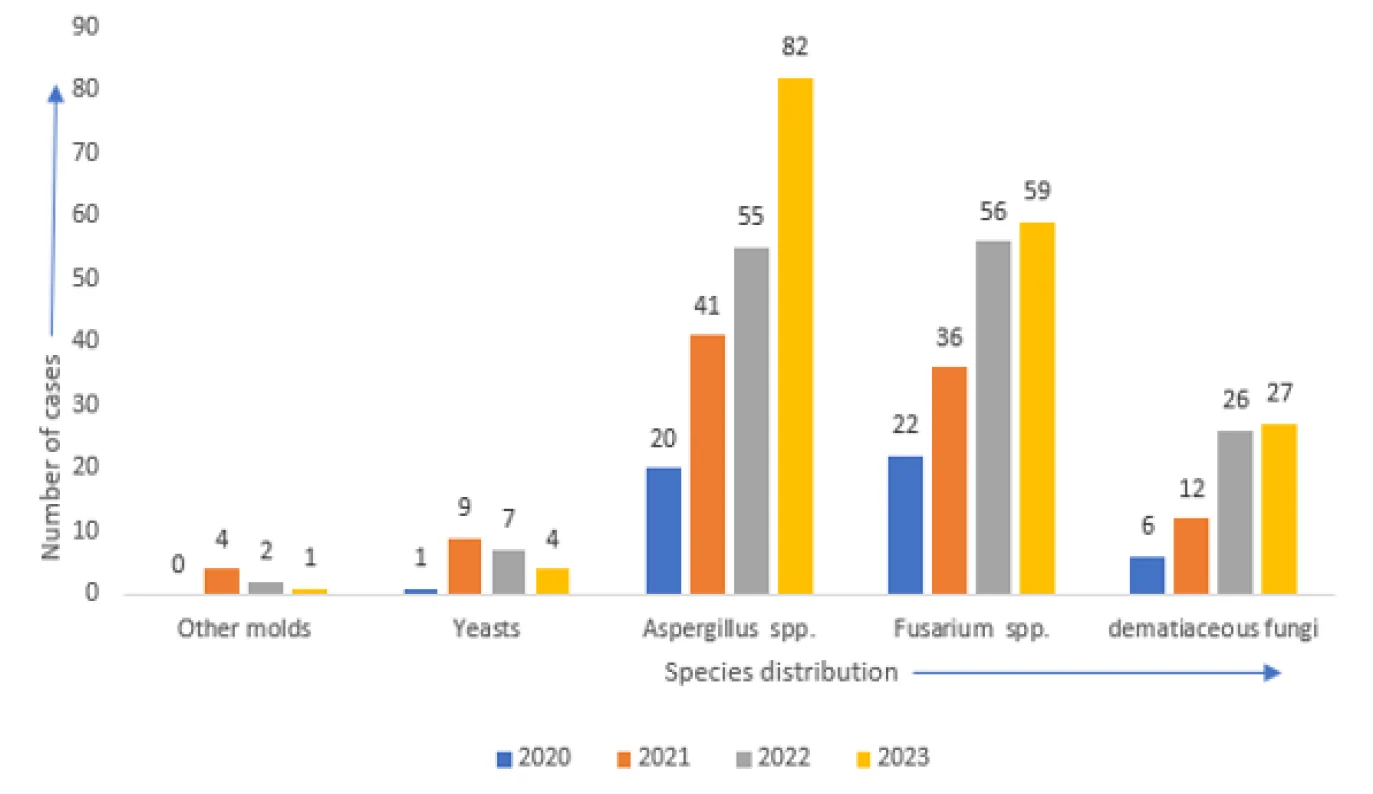
Year-wise distribution of fungal agents isolated from corneal samples.

### In vitro antifungal susceptibility testing

Among the 470 culture-positive cases, only 150 cultures could be revived from the mycology repository at the National Culture Collection of Pathogenic Fungi, PGIMER, and AFST against the selected antifungals. Susceptibility profiles are summarized in supplementary Tables S1 and S2. A total of 98 *Aspergillus* spp., which were tested, demonstrated low MICs to triazoles, with VRCZ, ITRA, and PSCZ showing activity in 98.97% (n=97), 97.95% (n=96), and 81.63% (n=80) of isolates, respectively, with MICs ranging from 0.25 to 8 µg/ml for VRCZ and PSCZ, and 0.06-1µg/ml for ITRA. In contrast, polyenes, with NTM and AMB, showed low MICs in 22.44% (n=22/98) and 44.89% (n=44/98) of isolates, respectively. The MIC ranges were 2–32 µg/ml for NTM and AMB 0.5-16 µg/ml. Among the other newer promising agents, namely LLCZ, TFH, KTZ, and MCZ, were tested against all 98 *Aspergillus* spp. and demonstrated a good *in vitro* activity, with low MICs observed in 97.95% (n=96), 94.89% (n=93), 74.48% (n=73), and 76.53% (n=75), respectively. The MIC ranges for LLCZ, TFH, KTZ, and MCZ were 0.001-0.25 µg/ml, 0.12-0.5 µg/ml, 1-16 µg/ml, and 2-16 µg/ml, respectively.

Among the 40 *Fusarium* spp. tested, triazoles demonstrated low MICs in 92.5%, 80%, and 72.5% of the isolates to VRCZ (n=37), ITRA (n=32), and PSCZ (n=29), respectively, with MIC ranges of 0.12–16 µg/ml for VRCZ and PSCZ and 0.06–16 µg/ml for ITRA. Among polyenes, NTM and AMB showed low MICs in 60% (n=24) and 95% (n=38) of the isolates, with MIC ranges of 0.5–16 µg/ml and 0.06–4 µg/ml, respectively. Notably, the other antifungals, LLCZ, TFH, KTZ, and MCZ, showed low MICs in 97.5% (n=39), 92.5% (n=37), 87.5% (n=35), and 80% (n=32) of isolates, with MIC ranges of 0.001–0.016 µg/ml, 0.12–8 µg/ml, 0.12–16 µg/ml, and 0.06–16 µg/ml, respectively.

The 12 dematiaceous fungal isolates revealed that VRCZ and PSCZ exhibited low MICs in 75% (n=9) of isolates each, while ITRA showed low MICs in 58.33% (n=7) of the isolates. The respective MICs ranged from 0.5–16 µg/ml for VRCZ, and 0.25–16 µg/ml for PSCZ and ITRA. Among the polyenes, AMB demonstrated low MICs in 58.33% (n = 7) of isolates, with a range of 0.5–8 µg/mL, whereas NTM exhibited high MICs across all isolates. Among other antifungals, LLCZ and MCZ demonstrated low MICs in 58.33% (n=7) and 50% (n=6) of cases with values of 0.001–0.5 and 0.25–16 µg/mL, respectively. Moreover, TFH and KTZ demonstrated low MICs in 66.66% (n=8) of cases with MIC ranges of 0.12–2 and 0.5–16 µg/ml, respectively.

**Table 3:** Antifungal susceptibility profiles (Median MIC, MIC range, and their Geometric Mean in µg/ml) for clinical isolates of *Aspergillus* spp., *Fusarium* spp., and *Dematiaceous* Fungi.

Sr. No.	Antifungals tested	*Aspergillus spp.*			*Fusarium spp.*			Dematiaceous fungi		
		Median MIC (µg/ml)	Range (µg/ml)	GM (µg/ml)	Median MIC (µg/ml)	Range (µg/ml)	GM (µg/ml)	Median MIC (µg/ml)	Range (µg/ml)	GM (µg/ml)
1	VRCZ	0.25	0.25-8	0.65	0.5	0.12-16	0.57	0.5	0.5-16	0.85
2	ITRA	0.5	0.06-1	0.60	1	0.06-16	0.73	0.5	0.25-16	0.58
3	PSCZ	0.5	0.25-8	0.47	0.5	0.12-16	0.50	0.25	0.25-16	0.37
4	NTM	8	2-32	8.00	4	0.5-16	3.13	32	8-32	25.99
5	AMB	4	0.5-16	3.16	0.5	0.06-4	0.12	2	0.5-8	1.88
6	LLCZ	0.001	0.001-0.25	0.001	0.001	0.001-0.016	0.0012	0.008	0.001-0.5	0.015
7	MCZ	2	2-16	1.98	2	0.06-16	0.89	2	0.25-16	1.41
8	KTZ	2	1-16	1.89	0.5	0.12-16	0.61	1	0.5-16	1.25
9	TFH	0.12	0.12-0.5	0.12	0.12	0.12-8	0.20	0.5	0.12-2	0.46

MIC: Minimum Inhibitory Concentration, GM: Geometric mean, VRCZ: Voriconazole, ITRA: itraconazole, PSCZ: Posaconazole, NTM: Natamycin, AMB: Amphotericin B, LLCZ: luliconazole, MCZ: Miconazole, KTZ: Ketoconazole, TFH: terbinafine-hydrochloride

## Discussion

In India, FK is a great concern with newer fungi being added to the list, and their susceptibility patterns remaining unknown [9]. This study analyzed the FK etiology spectrum, risk factors, and susceptibility patterns, contextually comparing with existing literature.

The predominant causative agent reported in this study was *Aspergillus* spp., which correlated with reports of Ghosh et al., Tawde et al., and multiple other studies [8], [10,16-20]. *Fusarium* spp. have been reported as the second predominant agent of FK in the present study, which is concordant with other published studies, yet discordant with studies of Northern Thailand, Australia, and South India [8,15,17,21-25].

Recent trends in FK have shown a rise in dematiaceous fungi, posing notable diagnostic and therapeutic challenges due to limited reported literature. Dematiaceous fungi causing FK were reported to be 31.3% by Sengupta et al., and 21.9% by Ghosh et al., studies that also underscore the unclear breakpoints in CLSI guidelines [10,16]. In tropical regions, dematiaceous fungi are responsible for 8-17% of all FK cases [26, 27].

Trauma-induced infections by vegetative matter were an important etiological factor of FK, a result augmented by findings of a study performed by Tawde et al., which indicated that trauma from vegetative matter was a major risk factor (65.48%) for *Aspergillus* FK [8].

The AFST helps optimize antifungal choices and improve patient outcomes. The ECV identifies potential resistance by distinguishing wild-type from non-wild-type strains, while clinical breakpoints predict treatment success by classifying organisms as susceptible or resistant based on clinical and pharmacological data. For dematiaceous fungi, there are no established breakpoints in CLSI recommendations; only ECVs are available for a limited number of organisms [14]. The ECVs are available in *Aspergillus* and *Fusarium*, against the antifungals VRCZ, ITRA, PSCZ, and AMB, but not for NTM, KTZ, LLCZ, MCZ, and TFH; hence, ECVs were relied upon for the latter set of antifungals. The subsequent AFST interpretative terms used were “high MIC” and “low MIC” instead of resistant and susceptible [28].

Among the azoles, one *A. flavus* isolate showed a high MIC against VRCZ. Azole resistance in *A. flavus* is primarily associated with mutations in the cyp51A and cyp51C genes, which encode lanosterol 14α-demethylase, the target of azole drugs [29]. Overexpression of cyp51 genes and efflux pumps, MDR and ABC transporters also contribute to azole resistance [29, 30].

Results of the present study confirmed those of a study performed by Manikandan et al, which reported an MIC range of 0.25-1 µg/ml for ITRA and 0.25-4 µg/ml for VRCZ against *A. flavus* [31]. Louisa Lu et al. reported a median MIC of 0.5 µg/mL for *Aspergillus* spp. against VRCZ, which is comparable to the finding of the present study (0.25 µg/mL) [32]. Other relevant studies conducted by Prajna et al. reported MIC_50/90_ for VRCZ 0.25/0.5 µg/ml, ITRA 0.125/0.25 µg/ml, and PSCZ 0.125 µg/ml against *Aspergillus* spp., and Öz Y et al. observed MICs of 1 µg/ml against VRCZ, 0.5 µg/mL against ITRA, and 0.12–0.25 µg/ml against PSCZ for *A. flavus* [33,34].

The AFST of *Fusarium* spp. in the present study yielded an MIC range of 0.12-16 µg/ml for VRCZ, comparable to that of a study conducted by Lu et al., who reported 0.25-16 µg/ml [32]. The study carried out by Manikandan reported VRCZ MIC_50/90_ of 4/8 µg/ml and ITRA MIC_50/90_ of 32 µg/ml [31]. P. Dallé da Rosa et al. reported a MIC range of 1–32 µg/mL for VRCZ and 2–128 µg/mL for ITRA, and Huang et al. reported a VRCZ MIC range of 2-16 µg/mL against *Fusarium* spp. [35,36]. Among dematiaceous fungi, the low MICs of VRCZ, PSCZ, and ITRA are comparable to those reported in a study conducted by H. Zheng et al., which were 0.06–0.25 µg/ml for VRCZ, 0.125–0.25 µg/ml for ITRA, and 0.03–0.06 µg/ml for PSCZ [37].

The LLCZ, a promising antifungal also tested in the present study, exhibited excellent in vitro activity. *Aspergillus* spp. showed low MICs, which is consistent with the results of previous studies; Omran et al. reported an MIC range of 0.004–0.062 mg/m, Furnica et al found an MIC_50/90_ of 0.002/0.015 µg/ml, and a USA study demonstrated a 0.001 µg/mL MIC [38-40]. Results of LLCZ against *Fusarium* showed a low MIC range of 0.001–0.004 µg/ml with a GM of 0.0012 µg/ml, comparable to a study conducted in Taiwan reporting an MIC of 0.031–0.25 µg/ml with a GM of 0.04 µg/ml [36]. Meanwhile, on the same note, Todokoro et al. reported MIC_50/90_ of 0.03/0.06 µg/ml [11]. The LLCZ against dematiaceous fungi exhibited low MICs in 58.33% of the isolates, conceivably the first *in vitro* susceptibility report of dematiaceous fungi causing FK.

The AFST results of KTZ and MCZ are also comparable to those of multiple studies. Hassan et al. reported low KTZ MICs in 29% of *Fusarium* isolates, while Xie et al. found low MICs in 22.2% of *A. flavus* and 82.4% of *F. solani* for MCZ, which are similar to the results of the present research [41,42]. Other relevant studies performed by Manikandan et al. reported MIC_50/90_ of 16 µg/ml for the *Fusarium* spp. and ¼ µg/ml for *A. flavus*, and Pujol et al. reported strong *in vitro* activity against *Fusarium* spp. [29,43].

Among polyenes, NTM showed an MIC range of 2-32 µg/ml against *Aspergillus* spp., consistent with previous studies; Prajna et al. reported an MIC_50/90_ of 32/>32 µg/ml, Manikandan et al. reported an MIC of 16-32 µg/ml, Stephen et al. reported an MIC of 1–64 µg/ml, and D.U. Gajjar reported an MIC of 8–32 µg/ml. The NTM, although preferred for FK, is limited by poor corneal penetration [3,31,33,44].

In this study, 60% of *Fusarium* spp. showed low MICs (0.05–16 µg/ml) against NTM, differing from the results of a study performed by Manikandan et al. reporting an MIC of 16–32 µg/ml. Hassan et al. also contradicted this finding by reporting that only 1.5% of *Fusarium* isolates were susceptible to NTM [31,41]. The NTM against dematiaceous isolates exhibited high MICs in the present study, comparable to the study conducted by D. U. Gajjar et al. [44]. No correlation was observed between NTM susceptibility and clinical outcomes.

In the present study, AMB exhibited a low MIC range of 0.5-16 µg/ml in 44.89% of *Aspergillus* spp., comparable to the studies performed by Xie et al., reporting low MICs in 66.7% of *A. flavus*, and Manikandan et al., reporting an MIC of 0.25–8 µg/ml [31, 42].

For *Fusarium* spp., AMB demonstrated an MIC range of 0.12–4 µg/ml. Supporting this, Ozdemir et al. reported 95% isolates with low MICs, and Xie et al. reported 82.4% AMB-susceptible *Fusarium solani* and 74% of AMB-susceptible *Fusarium oxysporum* [42,45]. The AMB showed low MICs in 58.33% of our dematiaceous isolates, with an MIC range of 0.5–8 µg/mL, a result concordant with those of a study conducted by D. U. Gajjar et al., reporting MICs of 0.25–32 µg/mL for Curvularia lunata and 0.5–2 µg/mL for *Exserohilum rostratum* [44].

The TFH demonstrated notable efficacy against *Aspergillus* spp. with a median MIC of 0.12 µg/ml. Owing to limited data, the only study available, Xie et al., reported TFH as effective against *Aspergillus* spp. [42].

In FK, host immunity plays a key role in controlling infection; immunocompromised individuals are more susceptible to severe, progressive disease [46]. Delayed diagnosis further worsens outcomes, allowing the fungus to invade deeper corneal layers, reducing treatment efficacy, and increasing the risk of vision loss [47]. Early recognition and prompt antifungal therapy are critical for a better prognosis [48].

Limitations of this study included incomplete patient data and outcomes, low isolate revival rates, as well as the absence of broth microdilution cut-offs for clear susceptibility classification.

## Conclusion

*Aspergillus flavus* was the predominant FK pathogen in our North Indian tertiary care centre. The VRCZ is highly effective in the susceptibility pattern. The LLCZ and TFH emerged as promising antifungals, showing strong potential for future clinical use in the management of FK.
